# Glomerulonephritis with Crescents in Children: Etiology and Predictors of Renal Outcome

**DOI:** 10.5402/2011/507298

**Published:** 2011-10-30

**Authors:** K. Alsaad, N. Oudah, A. Al Ameer, K. Fakeeh, A. Al Jomaih, A. Al Sayyari

**Affiliations:** ^1^Department of Pathology, King Abdulaziz Medical City, P.O. Box 22490, Riyadh 11426, Saudi Arabia; ^2^Department of Pediatrics, King Abdulaziz Medical City, P.O. Box 22490, Riyadh 11426, Saudi Arabia; ^3^Department of Medicine, College of Medicine, King Saud Bin Abdulaziz University for Health Sciences, P.O. Box 22490, Riyadh 11426, Saudi Arabia; ^4^Department of Medicine, King Saud Bin Abdulaziz University for Health Sciences, P.O. Box 22490, Riyadh 11426, Saudi Arabia

## Abstract

*Objective*. To investigate the clinicopathological features and outcome of glomerulonephritis with crescents among Saudi children. *Method*. This is a retrospective study of cases of crescentic glomerulonephritis (CrGN) seen over a 9-year period. Histological features and renal function were recorded. *Results*. Thirty-seven cases were enrolled. The mean percent of glomeruli with crescents was 39% (±19). Lupus nephritis (LN) was the commonest etiology (54.1%). At presentation, the serum creatinine (SCr) was 218.2 (±174.3) umol/l, and 57.1% of the cases had nephrotic range proteinuria. By the end of the observation period, SCr dropped to 81.0 (±67.7) umol/l (*P* = 0.001). Worsening renal function was associated with younger age (*P* = 0.002), non-LN etiology (*P* = 0.01), more crescents (*P* = 0.019), and ATN (*P* = 0.05). At the end of the followup, more patients in the LN group were dialysis-free (*P* = 0.017) and had improved renal function (0.01) than in the non-LN group. Using multivariate analysis, the only independent factor found to predict need for dialysis or change in SCr level was percent of globally sclerosed glomeruli (*P* = 0.034). *Conclusion*. LN is the main cause of CrGN in our cohort of children. The LN group had less globally sclerorsed glomeruli and better renal prognosis than the non-LN group.

## 1. Introduction

Of all renal biopsies done in the pediatric population, 5% to 15% are due to crescentic glomerulonephritis [1, 2]. This variation in frequency is most likely due to differences in the indication criteria. Use for the biopsy.

The criteria for the number of affected glomeruli required for the diagnosis of crescentic glomerulonephritis vary from 20 to 75% in various pediatric studies [3]. The reports on crescentic glomerulonephritis in children are very few and most of these reports are over a decade old [2, 4–6]. Most of these reports suggest that the comments cause for crescentic glomerulonephritis in children is postinfectious glomerulonephritis and that the prognosis is generally poor. 

There are no previous reports on crescentic glomerulonephritis or lupus nephritis in Saudi children. We undertook this study to assess the etiology, presentation, and outcome in Saudi children with biopsy-proven crescentic glomerulonephritis.

## 2. Methods

This is a retrospective descriptive study. All the renal biopsies in children below the age of 18 years done over a 9-year period (2001–2009) which showed crescents in >20% of the glomeruli were included. All biopsies were reviewed by light microscopy, immunofluorescence, and electron microscopy and reviewed by a North American trained nephropathologist blinded to the patient outcome. The etiology was ascertained by clinical assessment, serological tests (including anti-DNA, ASO titer, anti-GBM, ANCA, C3, and C4) and histological criteria. The diagnosis of SLE in the patients with this entity was made based on the American Rheumatologic Association criteria of SLE [7]. The histological findings recorded included the number of glomeruli with crescents present, global and segmental glomerular sclerosis, acute tubular necrosis (ATN) and interstitial infiltrate. Lupus nephritis was diagnosed and classified based on the ISN/RPS classification [8]. The activity and chronicity, in cases of lupus nephritis, were scored based on previously described methods [9].

The serum creatinine levels and 24-hour proteinuria and serological tests were recorded at the time of biopsy and at the end of followup period. The primary outcome investigated was renal outcome. 

Descriptive statistics were generated. The baseline and end of followup means was compared using two-tailed paired *t* test and non-paired continuous data was compared using independent *t* test and one way ANOVA for comparing multiple continuous data Significance of changes for categorical data (e.g., gender, etiology) was assessed using Chi-Square test or Kruskal Wallis. The risk and prognostic factors for different variables were analyzed by linear multivariate regression when the dependent factor is continuous and by logistic multivariate regression when it is categorical. A *P* value < 0.05 was considered significant. All statistical analyses were carried out using the SPSS 17 software package. The local Institutional Review Board approved the study.

## 3. Results

Thirty-seven children had biopsyproven crescentic glomerulonephritis. The mean followup period from the time of biopsy was 30.9 (±22) months. 

The causes of crescentic glomerulonephritis was lupus nephritis in 20 (54.1%), postinfectious glomerulonephritis in 6 (16.2%), immune complex glomerulonephritis in 5 (13.5%), pauci immune glomerulonephritis in 3 (8.1%), and others in 3 (8.1%). The mean age in the whole group, was 13.2 (± 5.6) years, 15.8 (± 3.0) in the lupus nephritis group and 9.4 (± 4.7) years in the nonlupus group (*P* = 0.000). The females comprised 75.7% of the whole group and 80% of the lupus nephritis group (*P* = NS). (Table 1)

At the time of biopsy, 68.20% had acute renal failure, 12.5% required dialysis, 95.6% had proteinuria and 52.2% had nephrotic range proteinuria, (Table 2). There were no significant differences between the lupus nephritis and nonlupus nephritis groups in the prevalences of acute renal failure or proteinuria. However, there were over three times as many patients in the non-lupus nephritis group who required dialysis at biopsy (Table 2). In the group as a whole, the serum creatinine and 24-hour urine excretion, at the time of biopsy, were 218.2 (±174.3) umol/l and 5.1 (±6.4) gms, respectively. By the end of the follow-up period, these values dropped 81.0 (±67.7) umol/l (*P* = 0.001) and 2.7 (±6.9) gms (*P* = 0.008), respectively.

At the end of the followup period, the lupus nephritis group fared better than the nonlupus nephritis group in that less patients required dialysis (*P* = 0.017) and more patients showed improved renal function (*P* = 0.01). However, there was no significant difference in end of follow-up proteinuria (*P* = 0.5) (Table 3) 

Among the lupus nephritis group, the mean activity score was 11.4 (±4.3) and the mean chronicity score was 3 (±1.2).  Table 4 shows that there were no significant differences in the histological findings between the lupus and nonlupus groups except that there were more globally sclerorsed glomeruli in the non-lupus group (*P* = 0.05). Although the non-lupus group had higher percentage of glomeruli with crescents, higher interstitial fibrosis/atrophy, acute tubular necrosis, and vasculopathy than the lupus nephritis group, none of these reached significant levels (Table 4). 

In the group as a whole, the factors associated with worsening renal function were younger age (*P* = 0.002), being non-lupus nephritis (*P* = 0.01), higher percent of glomeruli with crescents (*P* = 0.019), presence of more ATN on biopsy (*P* = 0.05), and more vasculopathy (*P* = 0.02) (Table 5). 

We removed the extra right delimiter after the highlighted part. Please check.

Using logistic multivariate analysis, the only independent factors found to predict need for dialysis were percent of globally sclerorsed glomeruli (*P* = 0.034). Baseline proteinuria or SCr, gender, number of glomeruli with crescents, on the other hand, did not impact prognosis. Using linear regression multivariate analysis, SCr, protein excretion, and activity score at biopsy did not influence change in SCr or final SCr during the followup period. 

No correlation as found between the changes in serum creatinine over the observation period and any of the following parameters: baseline SCr or proteinuria but there was a significant correlation with % of glomeruli with crescents (*r* = 0.98, *P* = 0.03) (Table 6).

## 4. Discussion

There are very few papers on crescentic glomerulonephritis in children. This is the first paper describing Saudi children with condition. One of the most striking differences we noted from other reports is in the etiology of crescentic glomerulonephritis. In our group, lupus nephritis and postinfectious GN contributed 54.1% and 16.2% of the cases, respectively. In a recent report on Indian children, lupus nephritis contributed only 9.1% with the main etiology being post infectious GN (36.8%) [1].  Table 7 compares our findings to reports from IUSA [4], India [1], France [5], and UK [6] on crescentic glomerulonephritis in the pediatric group. The most striking differences can be seen in the etiology and frequency of renal loss. In our group, the etiology of the glomerulonephritis was lupus nephritis in over 50% of the cases, whereas in the other groups less than 10% of the cases were due to lupus nephritis. We also observed a better renal outcome in our patients. This may simply be related to the less severe baseline renal impairment (Table 7). 

In our study, the lupus nephritis group had a better prognosis despite higher baseline serum creatinine. More patients in this group were dialysis-free by the end of the observation period (*P* = 0.017) and showed improvement in renal function by the end of the observation period (0.01). This might reflect the less percent of glomeruli with crescents and those with global sclerosis in the LN group. Similar good renal outcome was reported by others [10–12] (Table 8). In one study, [10] none of the children with lupus nephritis required dialysis which is similar to our finding (Table 8). Even the patients with Class IV lupus nephritis showed significant improvement in renal function [10]. Like us Bogdanović et al. found that gender and baseline renal function was not associated with worse renal function. However, unlike us, they found that that the presence of nephrotic syndrome was associated with worse renal prognosis. 

In a recent study on adults with crescentic lupus nephritis, the renal prognosis was also found to be good with only 4.3% developing end-stage renal failure at the end of a mean follow up period of 56 months [13]. 

Frequency and type of renal involvement in SLE is different in different ethnic groups being, for example, higher in Afro-Americans and Hispanics that Caucasians in USA [14]. Moreover, in Afro-Americans and Hispanics, the prognosis of LN seems to be worse than in Caucasians [14]. Scanty reports for KSA would suggest that there is a high prevalence of LN among Saudi patients with SLE [15]. 

## 5. Conclusion

In Saudi children, the commonest cause of crescentic glomerulonephritis by far is lupus nephritis. The factors associated with worsening renal function were younger age (*P* = 0.002), higher percent of glomeruli with crescents (*P* = 0.019), and the etiology being due to non-lupus nephritis (*P* = 0.01). The other factors constitute more evidence of ATN and vasculopathy (*P* = 0.02) on histology (*P* = 0.05). Others have also found that pathologic features associated with a poor prognosis included predominance of large crescents (*P* = 0.004), global glomerular sclerosis, interstitial fibrosis and tubular atrophy [4]. 

## Figures and Tables

**Table 1:** Comparing Lupus nephritis with other forms of crescentic GN (demographic and clinical data).

	All (*n* = 37)	LN (*n* = 20) (mean ± Std)	Non LN (*n* = 17) (mean ± Std)	*P*
Females (%)	57.5%	80%	70.6%	0.5
Age (years)	13.2 (±5.6)	15.8 (±3.0)	9.4 (±4.7)	0.000
SCr at biopsy (umol/l)	218.2 (±174)	243.7 (±178.1)	173.5 (±169.2)	0.38
SCr at follow up (umol/l)	81 (±67)	74.4 (±19.3)	94.3 (±119.1)	0.54
Baseline proteinuria (gms)	5.1 (±6.4)	5.4 (±7.7)	4.6 (±3.9)	0.8
Last proteinuria (gms)	2.7 (±6.9)	3.5 (±8.6)	1.5 (±2.3)	0.5
% Change in SCr	−28.4 (±48)	−44.7 (±37.6)	−7.1 (±53.8)	0.06
Change in proteinuria	−16.7 (±12.8)	−11.3 (±132.3)	−24.6 (±129.3)	0.8

**Table 2:** Baseline renal details.

	All	lupus	Nonlupus	*P*
Acute renal failure	68.20%	71.40%	50.00%	0.30
Needed dialysis	12.50%	8.30%	27.30%	0.04
% with proteinuria	95.60%	92.90%	100%	0.40
% with nephrotic proteinuria	52.20%	50.00%	55.60%	0.80

**Table 3:** Final renal details.

	At follow-up		
	Lupus	Nonlupus	*P*
Dialysis dependent	0%	36.4%	0.017
Improved	100%	60%	0.01
Proteinuria	3.6 (±8.6)	1.5 (±2.3)	0.5
Change in SCr (umol/l)	(−) 44.7%	(−) 7.1%	0.06
Change in proteinuria (gms/24 hours)	(−) 11.3	(−) 24.6	0.8

**Table 4:** Comparing lupus nephritis with other forms of crescentic GN (histological findings).

	LN (*n* = 20) (mean ± Std)	Non-LN (*n* = 17) (mean ± Std)	*P*
No of glomeruli in the biopsy	24.9 (±11.4)	26.8 (±11.5)	0.6
Globally sclerorsed glomeruli (%)	3.9 (±5.1)	13.8 (±22.2)	0.05
Glomeruli with crescents (%)	36 (±13)	42 (±24)	0.35
% glomeruli with BM thickening	30%	17.6%	0.4
% glomeruli with mesangial cellularity	15%	17.6%	0.8
% glomeruli with mesangial matrix expansion	35%	35.3%	0.98
% glomeruli with Interstitial fibrosis/atrophy	5%	11.8%	0.4
% glomeruli with ATN	25%	41.2%	0.3
% glomeruli with s vasculopathy	0%	11.8%	0.1

**Table 5:** Factors predicting renal function.

	Renal function improved	Renal function worsened	*P*
Age in years	13.5 (±5.6)	8.5 (±1.3)	0.002
Globally sclerorsed (GS) glomeruli (%)	6.4 (±9.3)	9.3 (±14)	0.3
Percent of glomeruli with crescents	35 (±17)	59 (±18)	0.019
Percent change in proteinuria	−15.2 (13.8)	−8.3 (±12)	0.2
Female gender	85%	75%	0.6
Lupus etiology	70%	0%	0.01
Significant glomerular infiltrate	35%	75%	0.1
Significant mesangial cellularity	10%	25%	0.4
Significant mesangial matrix expansion	30%	25%	0.8
Significant interstitial fibrosis/atrophy	10%	0%	0.2
Significant acute tubular necrosis	25%	75%	0.05
Significant vasculopathy	5%	25%	0.02

**Table 6:** Correlation with % change in serum creatinine.

Factor	*R*	*P*
% Gloms with Crescents	0.98	0.03
Baseline SCr	0.4	0.20
Baseline Proteinuria at biopsy	0.4	0.20
% change in proteinuria	0.33	0.22

**Table 7:** Comparing our findings to 4 other reports on crescentic GN among children.

	Dewan et al. [1] (*n* = 22)	No author [4] (*n* = 50)	Jardim et al. [6] (*n* = 30)	Niaudet and Levy [5] (*n* = 41)	This paper (*n* = 37)
Mean age (years)	12.3	10.1	9.5		13.2
M : F ratio	1.9 : 1	1 : 1.5	1 : 1.3		1 : 1.4
Renal loss (required dialysis)	45.5	48%	30%		16.7%
Presence of hypertension	95.5%	51%	63.3%		55%
Presence of Proteinuria		78%			95.5%
Baseline renal function	Mean SCr 484	GFR < 66%	GFR < 30 in 73.3% (mean SCr 457)		Mean SCr 218
Lupus Nephritis (%)	9.1	18	2.4	3.3	54.5
Immune complex GN (%)	59	56	59.9	75.3	21.6

**Table 8:** Comparing our findings to 3 other reports on lupus nephritis among children.

	Hobbs et al. [10]	Bogdanovíc et al. [11]	Wong et al. [12]	This paper
Mean age (years)	14.5	14.5		15.8
M : F ratio	1 : 19		15 : 1	4 : 1
Class 4	33.3%	64%	53. −%	95%
Renal loss (required dialysis)	0%	80% had complete or partial remission	3.1%	0%
Nephrotic	57.1%	40%	60%	50%
Baseline renal function	33.3% Dx; SCr 237	20% had renal impairment		SCr 244
